# Microstructure characterization of BaSnO_3_ thin films on LaAlO_3_ and PrScO_3_ substrates from transmission electron microscopy

**DOI:** 10.1038/s41598-018-28520-9

**Published:** 2018-07-06

**Authors:** Hwanhui Yun, Koustav Ganguly, William Postiglione, Bharat Jalan, Chris Leighton, K. Andre Mkhoyan, Jong Seok Jeong

**Affiliations:** Department of Chemical Engineering and Materials Science, University of Minnesota, Minneapolis, Minnesota 55455 USA

## Abstract

Detailed microstructure analysis of epitaxial thin films is a vital step towards understanding essential structure-property relationships. Here, a combination of transmission electron microscopy (TEM) techniques is utilized to determine in detail the microstructure of epitaxial La-doped BaSnO_3_ films grown on two different perovskite substrates: LaAlO_3_ and PrScO_3_. These BaSnO_3_ films are of high current interest due to outstanding electron mobility at ambient. The rotational disorder of low-angle grain boundaries, namely the in-plane twist and out-of-plane tilt, is visualized by conventional TEM under a two-beam condition, and the degree of twists in grains of such films is quantified by selected-area electron diffraction. The investigation of the atomic arrangement near the film-substrate interfaces, using high-resolution annular dark-field scanning TEM imaging, reveals that edge dislocations with a Burgers vector along [001] result in the out-of-plane tilt. It is shown that such TEM-based analyses provide detailed information about the microstructure of the films, which, when combined with complimentary high-resolution X-ray diffraction, yields a complete structural characterization of the films. In particular, stark differences in out-of-plane tilt on the two substrates are shown to result from differences in misfit dislocation densities at the interface, explaining a puzzling observation from X-ray diffraction.

## Introduction

Increased interest in BaSnO_3_ (BSO) has emerged in recent years due to its optical transparency in the visible range and attractive electronic transport properties, including high electron mobility, exceeding 300 cm^2^V^−1^s^−1^, in bulk single crystals at room temperature^1–3^. Recent studies have explored its potential use as a transparent conductive oxide in photovoltaic devices and as a channel material in field-effect transistors^4–11^. Advances in thin film growth have strengthened BSO’s relevance as a potential material in next-generation oxide electronics^12–15^, although the room temperature electron mobility in BSO thin films still lags that of single crystals^12–19^. Previous reports have elaborated a range of factors limiting mobility in epitaxial BSO films, primarily compensating charged defects such as point defects, impurities, and dislocations^13,15–18^. The latter are prevalent due to the current lack of a commercial substrate around the 4.116 Å BSO cubic lattice parameter, necessitating the use of substrates with significant lattice mismatch. These result in large defect densities, emphasizing the need for in-depth quantitative studies of microstructure in BSO thin films.

Although progress has been made, much remains to be understood regarding the microstructure of epitaxial BSO films. While the crystalline quality of an epilayer is dependent on various factors, such as growth methods, growth conditions, *etc*., the choice of substrate, *i*.*e*., the lattice mismatch, is particularly crucial^17–19^. The lattice mismatch between a BSO film and its substrate not only directly influences the formation of misfit dislocations (MDs) at the interface, but might also play some role in determining the density of threading dislocations (TDs), and the grain misorientations, throughout the film^19^. These are key factors for mobility-structure property relationships in BSO films. To date, most structural analyses in BaSnO_3_ thin films have been conducted *via* X-ray diffraction (XRD) to estimate a lattice constant, to confirm if they have a single phase crystal, or to examine their crystalline qualities^1–3,12–19^. While XRD analysis is very powerful for overall characterization of the *average* microstructure, information about critical *local* microstructural details can be obtained from alternative techniques. Transmission electron microscopy (TEM) is one such method, as it can provide detailed information about the microstructure of films through direct observation of local grain orientations, as well as MDs and TDs^20^. Conventional TEM (CTEM) techniques have thus been widely used to visualize dislocations and high-resolution scanning TEM (STEM) has been exploited to study characteristic atomic structures in many forms of thin films^13–15,17–19,21–25^. In particular, the structure at the film-substrate interface and characteristic defects in perovskite thin films, including BSO, have been studied recently^22,26,27^.

In this paper, we examine the microstructure of BSO films grown on LaAlO_3_ (LAO) and PrScO_3_ (PSO) substrates, with rather different lattice mismatch, using CTEM and annular dark-field-STEM (ADF-STEM). In particular, the two-beam condition in CTEM mode is utilized to evidence both in-plane grain twist and out-of-plane grain tilt in these BSO films with the degree of the grain twist being quantifiable from electron diffraction. Plan-view ADF-STEM images are also acquired to investigate the atomic structures and in-plane disorder in the films. Finally, the film-substrate interface is further explored using cross-sectional atomic-resolution ADF-STEM imaging. These results, in combination with complementary high-resolution XRD, enable us to elucidate the relationship between the microstructure of the films and the interface atomic structure, in particular elaborating the role of MDs in determining the very different out-of-plane tilts on the two substrates.

## Methods

DC sputter deposition from a 2-inch Ba_0.98_La_0.02_SnO_3_ target was performed to grow La-doped BSO films, using high pressure oxygen sputtering^28,29^. The target was synthesized by solid-state reaction of BaCO_3_, SnO_2_, and La_2_O_3_ powders (≥99.99% purity) in air at 1200 °C, followed by cold pressing and sintering at 1450 °C. Approximately 200-nm-thick, unbuffered, La-doped BSO films on LAO(001)_pc_ (8.7% mismatch) and PSO(001)_pc_ (2.4% mismatch) substrates were subsequently deposited at a DC current of 150 mA, substrate temperature of 900 °C, O_2_ pressure of 1.9 Torr, and growth rate of 7.5 Å/min. (The “pc” subscript here denotes pseudocubic notation for the rhombohedral LAO and orthorhombic PSO substrates). Post-growth cool-down was done in 600 Torr of O_2_. High-resolution XRD measurements were then carried out in both out-of-plane and grazing-incidence modes, using Cu *K*_α_ radiation in a Panalytical X’Pert Pro diffractometer. Electronic transport measurements in a van der Pauw geometry using In contacts were carried out in a Dynacool Physical Property Measurement System (Quantum Design, Inc.). These yielded 300 K electron density (*n*) and mobility (*μ*) of 3.7 × 10^20^ cm^−3^ and 49 cm^2^V^−1^s^−1^ for BSO/LAO, and 4.7 × 10^20^ cm^−3^ and 45 cm^2^V^−1^s^−1^ for BSO/PSO. It should be noted that these mobilities are quite similar, despite the rather different lattice mismatch in the two cases.

CTEM images and selected-area electron diffraction (SAED) patterns were obtained using an FEI Tecnai G2 F30 (S)TEM operated at 300 keV. The area selected by the selected-area aperture was about 0.7 μm^2^. ADF-STEM images were acquired using an aberration-corrected FEI Titan G2 60–300 (S)TEM operated at 200 keV. The convergence semiangle for the electron probe was 17.3 mrad. The low-angle ADF (LAADF) and high-angle ADF (HAADF) detector inner angles were in the range of 11–19 and 55–93 mrad, respectively. Plan-view TEM samples were prepared by mechanical polishing using Multiprep^TM^ (Allied High Tech Products, Inc.). The plan-view sample thickness was estimated to be in the range of 50–70 nm *via* the electron energy-loss spectroscopy log-ratio method, using a calculated mean free path of plasmon excitations in BSO of λ_p_ = 81 nm^30,31^. Cross-sectional TEM samples were prepared *via* a focused ion beam (FIB) lift-out method using an FEI Helios NanoLab G4 dual-beam FIB with a 30 kV Ga-ion beam. The samples were further polished at 2 kV and 1 kV to remove the majority of damaged surface layers. The thickness of the cross-sectional samples was in the range 40–50 nm.

### Data availability

The datasets generated during and/or analyzed during the current study are available from the corresponding author on reasonable request.

## Results and Discussion

**Figure 1:**
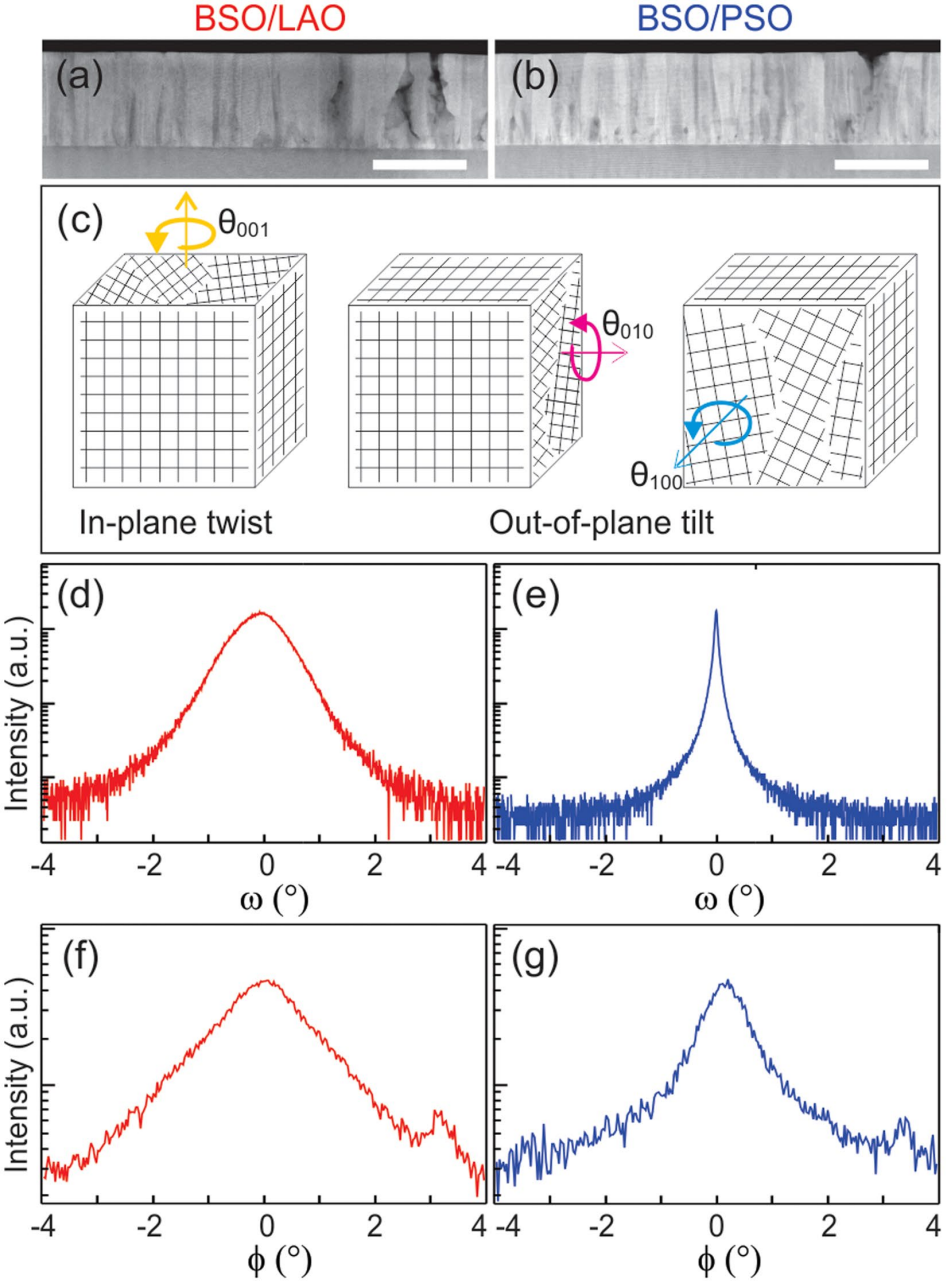
Cross-sectional HAADF-STEM images of BSO on (**a**) LAO and (**b**) PSO substrates. The scale bars in (**a**) and (**b**) are both 200 nm. (**c**) Schematics of the three basic forms of rotational disorder in these BSO films: The grain rotation around the [001] direction is denoted as “in-plane twist”, while those around the [010] and [100] directions are denoted as “out-of-plane tilt”. (002) X-ray rocking curves of BSO on (**d**) LAO and (**e**) PSO substrates, and grazing-incidence (020) X-ray rocking curves of BSO on (**f**) LAO and (**g**) PSO substrates.

The low-magnification cross-sectional HAADF-STEM images in Fig. 1(a,b) show the overall crystalline characteristics of the two epitaxial BSO films studied in this work. A columnar structure is clearly visible in the films on both substrates, pointing to the existence of rotational disorder, *i*.*e*., low-angle grain boundaries. Note that the exact thickness of the films was directly measured from the cross-sectional HAADF-STEM images to be 210 and 230 nm for BSO on LAO and PSO, respectively. A simple model illustrating the possible rotational disorder of grains in these films is provided in Fig. 1(c). Grains rotated with respect to one another around the [001] direction, *i*.*e*., the growth direction, result in what we term *in-plane twist*, while grain rotations around the [010] and [100] directions result in what we term *out-of-plane tilt*. Within this picture, even though reality may be more complicated, the rotational disorder in BSO films on LAO and PSO substrates can be analyzed from TEM data, and compared with XRD results.

To quantify the *average* rotational disorder in the films, XRD data were quantitatively analyzed first. Wide-angle out-of-plane and in-plane 2θ-ω coupled scans provide information on lattice parameters, strain state, and grain size in such films. The out-of-plane and in-plane lattice parameters of these BSO films were estimated to be 4.117 Å and 4.117 Å for the film on the LAO substrate, and 4.125 Å and 4.122 Å for the film on the PSO substrate, respectively. The X-ray rocking-curves (RCs), on the other hand, provide the average degree of rotational disorder. The in-plane and out-of-plane rotational disorders described in Fig. 1(c) are captured by the full-width-at-half-maximum (FWHM) of the ϕ and ω RCs, respectively. The measured out-of-plane ω RCs for BSO (002) and in-plane ϕ RCs for BSO (020) from both films are shown in Fig. 1(d–g). The XRD RCs from these two films are notably different. The ϕ RCs have worse signal-to-noise ratio than the ω RCs as a result of the film thickness being only 200 nm. More importantly, the FWHMs of the ω RCs for BSO on LAO and PSO are 0.925° and 0.045°, respectively, indicating that BSO on LAO has a much higher degree of out-of-plane tilt. The origin of such is not at all clear from XRD data alone. Additionally, the FWHMs of the ϕ RCs for BSO on LAO and PSO are 1.540° and 0.867°, respectively, indicating that BSO on LAO also has a higher degree of in-plane twist, although the difference on the two substrates is much smaller than for the out-of-plane case. The degree of in-plane twist in both samples is also bigger than that of the out-of-plane tilt. Again, the origin of such behavior is not clear from XRD data alone.

**Figure 2:**
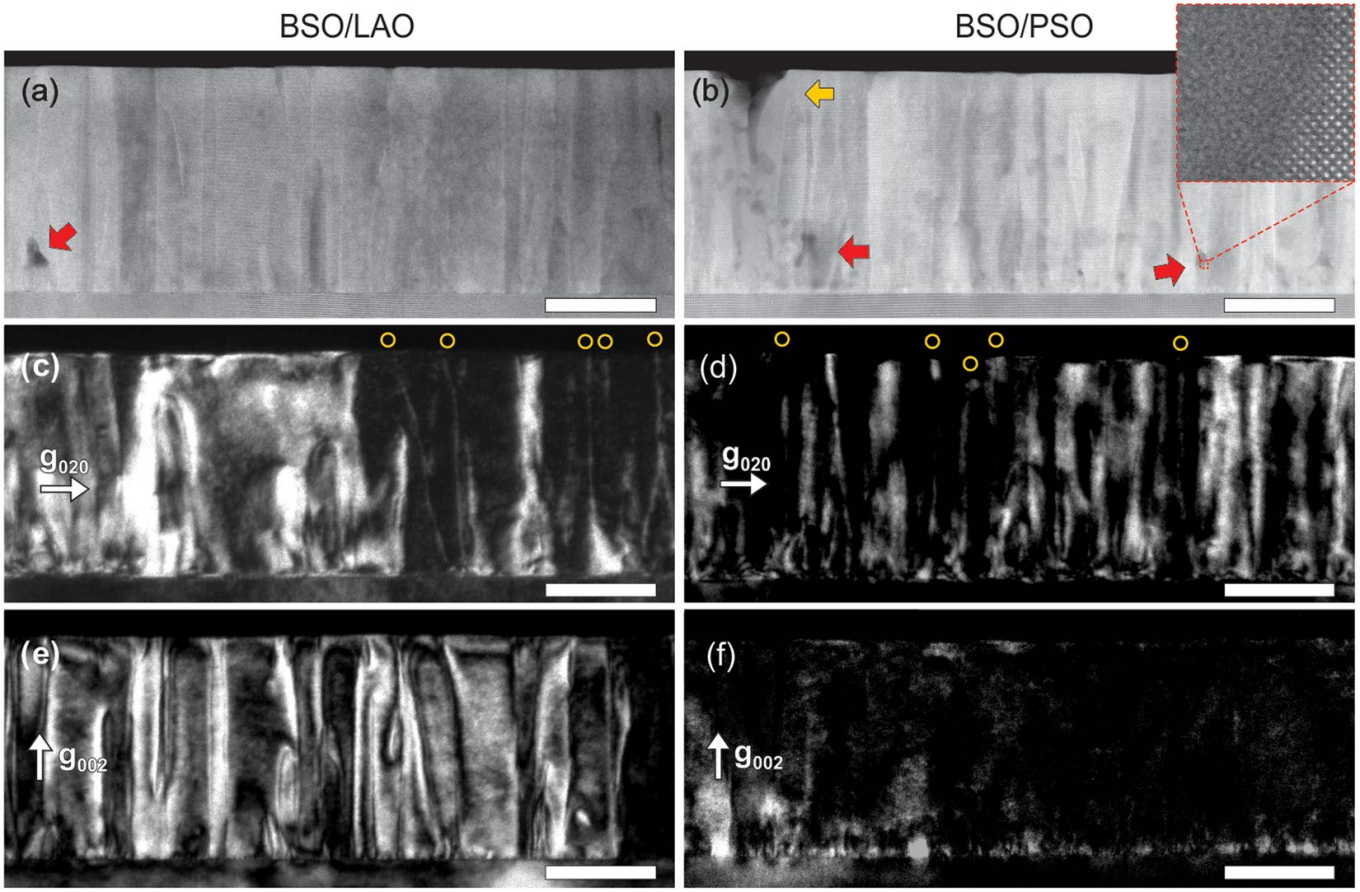
Cross-sectional STEM and CTEM images of BSO on LAO (left column) and PSO (right column). (**a**,**b**) HAADF-STEM images of BSO show *Z*-contrast with the presence of some voids (yellow arrow) and amorphous regions (red arrow). The inset in (**b**) shows a magnified image of the amorphous region next to crystalline grain. (**c**,**d**) TBDF images with **g** = 020 and (**e**,**f**) TBDF images with **g** = 002. Sharp line contrast is marked by the circles. Images were obtained from the same region in each sample, and all scale bars are 100 nm.

This rotational disorder in these BSO films can be characterized in greater detail using TEM. In Fig. 2(a,b), for instance, cross-sectional HAADF-STEM images of the BSO films on LAO and PSO clearly show vertical contrast from grainy columnar structures present in both films, in addition to some voids (yellow arrows) and amorphous regions (red arrows)^32^. The dislocations and rotational disorder in the grains can be well visualized by CTEM, using a two-beam condition known as two-beam dark-field (TBDF) imaging. A TBDF image is formed by a selected diffraction beam, thus the contrast in an image is sensitive to the corresponding reflection planes. When a dislocation is present and the selected reflecting planes are bent (*i*.*e*., nearby planes are strained), a bright contrast should be visible around the dislocation. Dislocations with a Burger’s vector **b** appear as line contrast in a TBDF image with reflection **g**, only if the combination of **b** and **g** does not satisfy the invisibility criteron: **g **· **b** = 0^20^.

Figure 2(c,d) show TBDF images of BSO films recorded with reflection **g** = 020, from the same regions shown in Fig. 2(a,b), respectively. Sharp lines running along the [001] direction are observed in both films (marked with yellow circles). These arise from TDs that, according to the invisibility criterion, are neither pure screw dislocations with **b** = a_BSO_[001], nor pure edge dislocations with **b** = a_BSO_[100]. In addition, regions with broad, band-like contrast are observed in these films. The in-plane twist of grains generate twist boundaries around which the (100) and (010) planes are misoriented (Fig. 1(c)). In a TBDF image with **g** = 020, the misorientation of the (020) planes creates contrast around the twist boundaries, as shown in Fig. 2(c,d). Therefore, grain boundaries that are not parallel to the electron beam direction appear with band-like contrast. They also overlap with the contrast from TDs in the TBDF images, because the TDs exist along the boundaries. Figure 2(e,f) show TBDF images recorded with the reflection **g** = 002. Here, significant bright band-like contrast is observed only in BSO on LAO. By applying the same analysis as performed above, it is thus deduced that this contrast originates from the out-of-plane tilt in the film (Fig. 1(c)). The (002) planes are strained around the tilt boundaries and generate the broad contrast. This observation of the out-of-plane tilt being dominant in BSO on LAO is consistent with the above XRD data (Fig. 1(d)).

It is found that the position and width of the band-like contrast regions in the TBDF image with **g** = 002 (Fig. 2(e)) do not correspond to the contrast observed in the TBDF image with **g** = 020 (Fig. 2(c)), even though the images were obtained from the same region. This implies that in-plane twist and out-of-plane tilt in these BSO films are not necessarily correlated. Additionally, an average grain width was estimated from the distance between observed low-angle grain boundaries in TBDF images with reflection **g** = 022, which mostly includes contrast from both **g** = 002 and **g** = 020 (data not shown here). The average grain width was evaluated to be 31.1 ± 11.1 nm for BSO on LAO and 30.8 ± 11.9 nm for BSO on PSO (from a total of 1 μm length in each sample), indicating that the films have insignificant differences in grain size, despite their significantly different lattice mismatch. It should be noted here that the actual grain widths should be slightly bigger than these values, since some of the grain boundaries are likely overlapped in these TBDF images.

**Figure 3:**
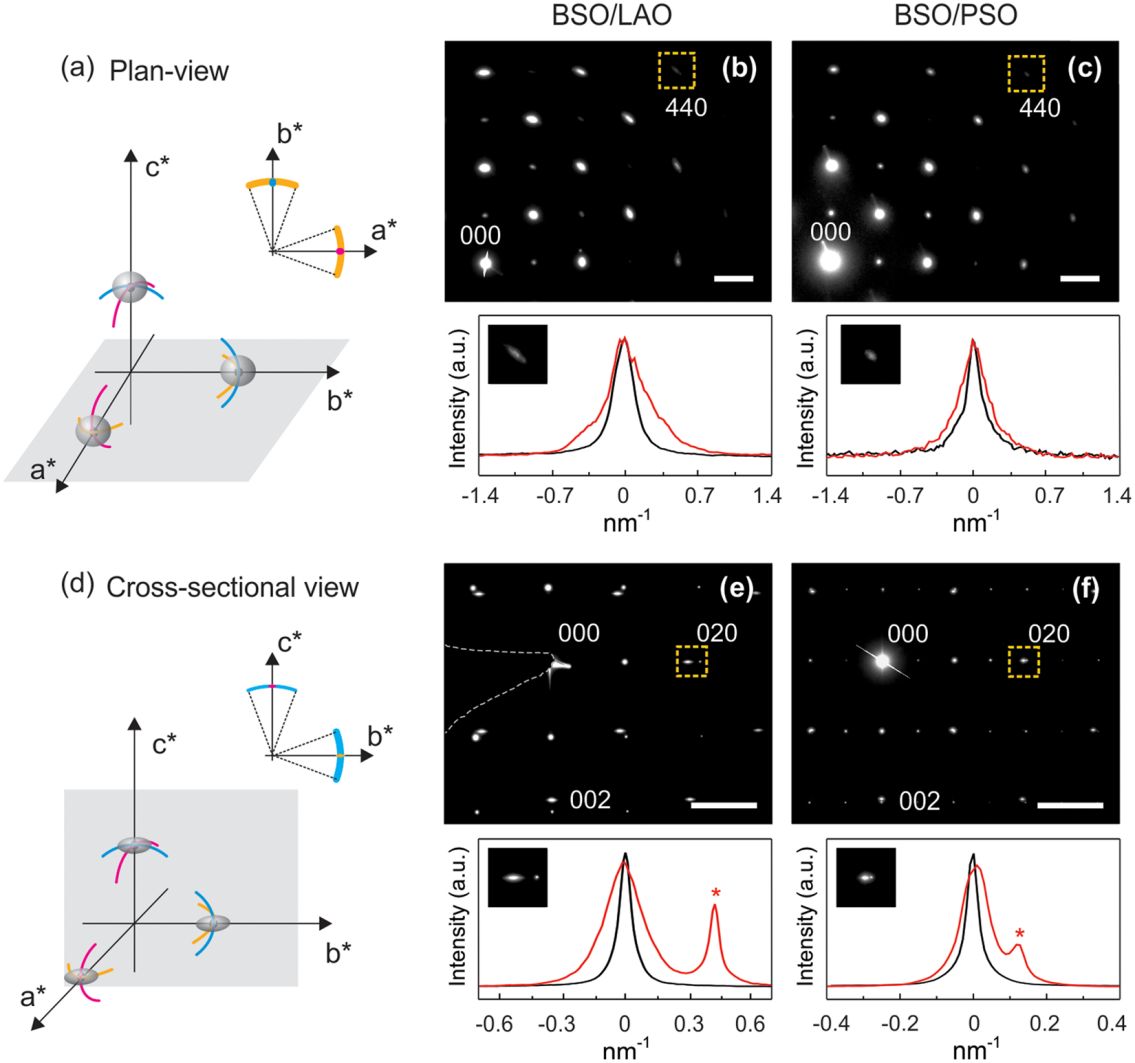
(**a**,**d**) Schematic illustrations of three-dimensional reciprocal space, where relrods are drawn according to the shape of grains in the TEM samples. The displacement of the reciprocal lattice due to in-plane twist and out-of-plane tilt is depicted by the yellow and blue/magenta lines, respectively. The Ewald sphere is drawn as the gray planes. The insets show expected SAED patterns. The experimental SAED patterns from BSO films on LAO and PSO are presented in (**b**,**e**) and (**c**,**f**), respectively. The scale bars in the SAEDs are 2 nm^−1^. The radial (black) and angular (red) profiles of the 440 diffraction spots in the plan-view SAEDs and the line profiles of 020 diffraction spots along the elongation (red) and perpendicular to the elongation (black) in the cross-sectional SAEDs are plotted below the corresponding SAEDs. The peaks marked by asterisks are from the substrates.

In principle, the degrees of in-plane and out-of-plane disorder in these films can be quantified using SAED patterns obtained from plan-view and cross-sectional samples. To assist in interpretation of such patterns, reciprocal space schematics for BSO films are provided in Fig. 3(a,d). The reciprocal lattice points are shown as finite shapes to represent relrods, which are determined by the shape of grains and the geometry of the TEM sample^20,33^. A SAED is formed when the Ewald sphere of the incident electron beam cuts through these relrods. In a plan-view sample, the electron beam propagates along the [001] direction, and, therefore, the Ewald sphere cuts through the relrods on the (a*b*) plane. When there is rotational disorder, the reciprocal lattice points deviate along the colored lines illustrated in Fig. 3(a): The yellow lines correspond to in-plane twist, whereas the blue/magenta lines signify out-of-plane tilt. Because the sample thickness is slightly larger than the average grain width, the relrod is predominantly determined by the grain width and has a broad disk shape slightly flattened in the (a*b*) plane^20,33^. As shown in the inset to Fig. 3(a), in plan-view SAED the in-plane twist results in an angular spread (yellow arc in the inset of Fig. 3(a)) of diffraction spots, while the out-of-plane tilt has insignificant effect on the deviation of diffraction spots.

Measured plan-view SAED patterns from BSO on LAO and PSO are presented in Fig. 3(b,c). Angular spread of the diffraction spots is clearly visible (in both films), indicating that in-plane twist indeed exists in both films. To quantify the extent of in-plane twist, angular and radial intensity profiles were obtained from 440 diffraction spots (see bottom panels of Fig. 3(b,c)). The degree of in-plane twist was evaluated as follows: (i) four 440 spots from three different regions of a film were analyzed and the results were averaged to minimize the effect of the TEM sample orientation and to have better statistics; (ii) the spread in the angular profile with respect to the radial profile was estimated by quadratic subtraction of the two FWHMs (assuming the peaks are Gaussian). The estimated angular spread was 0.31 ± 0.04 and 0.24 ± 0.03 nm^−1^, corresponding to in-plane twists of 1.8° ± 0.3° and 1.4° ± 0.2°, for BSO on LAO and PSO, respectively. Importantly, the values obtained from this analysis are thus similar to those measured from XRD, which were 1.540° and 0.867° for BSO on LAO and PSO, respectively. The discrepancy between the SAED and XRD results is likely due to the smaller sampling area in TEM.

Cross-sectional SAED patterns were also analyzed (see Fig. 3(d–f)). Here the Ewald sphere cuts through the (b*c*) plane, and the relrod shape is slightly different in a cross-sectional sample. The film thickness (along the [001] direction) is about 200 nm and the grain width (along the [010] direction) is about 30 nm. Therefore, the relrod has a thin disk shape^20,33^, with the disk being parallel to the (a*b*) plane, as in the plan-view sample. The cross-section of the relrod made by the Ewald sphere will create streaks in a SAED pattern. The out-of-plane tilt around the a* direction induces angular spread of diffraction spots (blue lines in Fig. 3(d)), whereas the out-of-plane tilt around the b* direction and the in-plane twist have an insignificant effect on the diffraction spot (magenta and yellow lines in Fig. 3(d)). In principle, this out-of-plane tilt can also be measured from the angular spread (discussed below). Figure 3(e,f) show the experimental cross-sectional SAED patterns obtained from BSO on LAO and PSO. As expected, the 020 diffraction spots from BSO are elongated along the b* direction in both films, which originates from the relrod.

**Figure 4:**
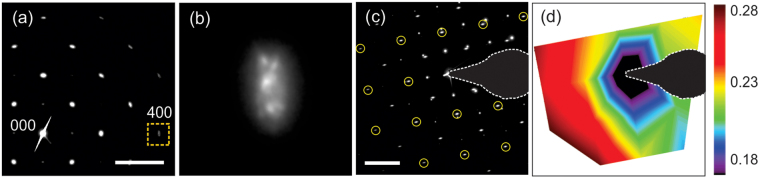
(**a**) Plan-view and (**c**) cross-sectional diffraction patterns of BSO on LAO. Scale bars are 4 nm^−1^ in (**a**) and (**c**). The 400 diffraction spot from (**a**) is magnified in (**b**). (**d**) Map of the elongations of diffraction spots, marked by the circles in (**c**), in the b* direction from BSO. The unit in (**d**) is nm^−1^.

To evaluate the extent of elongation of the 020 diffraction spot, the line profiles along the elongation, and those perpendicular to the elongation, were acquired (see the bottom panel of Fig. 3(e,f)). The extents of elongation were estimated to be 0.16 ± 0.04 and 0.06 ± 0.01 nm^−1^ for BSO on LAO and PSO, respectively. Assuming the grains have predominantly cylindrical shape, the width of the relrod is approximated as 2/d, where d is a grain width^20,33^. The half-width of relrods along the b* and c* directions should then be about 0.033 and 0.005 nm^−1^ for BSO films having a grain width of 30 nm and thickness of 200 nm. This indicates that the streak from the relrod cannot fully explain such large elongation of the 020 spot. In-plane microstrain in the film, however, specifically local in-plane lattice parameter variation, can lead to the elongation of diffraction spots in the b* direction. A plan-view diffraction pattern including many grains indeed evidences in-plane microstrain in the BSO film on LAO, as shown in Fig. 4(a,b). In the magnified 400 diffraction (Fig. 4(b)), scattered spots are indeed observed, in addition to the haze from the relrod, indicating the presence of microstrain in the film. The effect of in-plane microstrain was also observed on the map of the extent of elongation in Fig. 4(d). If the elongation were to originate only from the relrod shape, it would decrease with distance away from the 000 beam because of the curvature of the Ewald sphere. However, the elongation here actually increases in diffraction spots that are further away from the 000 beam, which provides additional evidence of microstrain in the film. Considering the film thickness and grain width of both films to be similar, the estimated elongation from both films indicates that BSO on LAO has more microstrain than BSO on PSO.

The angular spread in the cross-sectional SAED pattern resulting from the out-of-plane tilt is not very obvious, as shown in Fig. 3(e,f). When a film is fully relaxed along the out-of-plane direction, the 002 spot shows no effect of the misfit strain, and hence the amount of the out-of-plane tilt can be measured from the cross-sectional SAED pattern by comparing the angular profiles of 020 with respect to the beam spread in 002. The out-of-plane tilt is represented in the angular spread in the reciprocal lattice, which is indicated by the blue arc in the inset of Fig. 3(d). However, the SAED-based measurement of the out-of-plane tilt in the cross-sectional sample was not successful. The reason for this is four-fold: (1) Cross-sectional SAED includes a much smaller number of grains compared to the plan-view SAED. Furthermore, only the out-of-plane tilt around the electron beam direction is detected in the SAED data (in the simple model of Fig. 1(a), one of two out-of-tilts is visible). (2) The resolution of the SAED pattern also limits detection of out-of-plane tilt in the diffraction pattern when the degree of the tilt is too small (note that XRD data shows the out-of-plane tilt is smaller than the in-plane tilt). (3) Out-of-plane microstrain, linked to the above-mentioned in-plane microstrain, may be present in the film, changing the shape of the diffraction spots. (4) The accuracy of this measurement is further compromised with experimental uncertainties such as sample misorientation to the zone axis. This limitation in TEM diffraction pattern analysis can be relieved with information from ω X-ray RCs.

**Figure 5:**
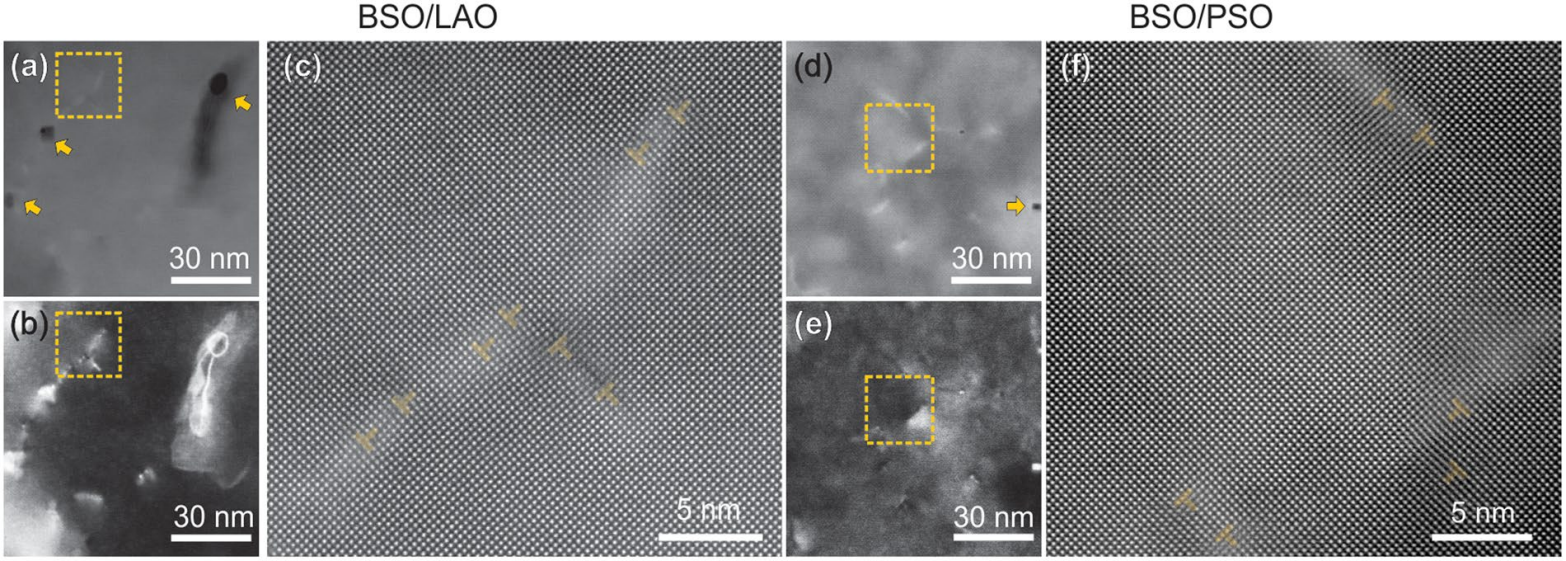
Plan-view ADF-STEM images of BSO on LAO (**a**–**c**) and PSO (**d**–**f**): (**a**,**d**) HAADF-, (**b**,**e**) LAADF-STEM, and (**c**,**f**) high-magnification HAADF-STEM images. The pairs of HAADF- and LAADF-STEM images were obtained from the same region. High-magnification HAADF-STEM images were obtained from the regions indicated by the boxes. Voids are marked with the arrows. Edge dislocations are marked with the dislocation symbols in (**c**) and (**f**).

The observation of predominant in-plane twist in BSO on LAO and PSO raises a question about how this grain twist affects the atomic structures of the films. For this, plan-view ADF-STEM images were acquired with different detector angles (Fig. 5). LAADF-STEM images clearly show bright strain contrast (see Fig. 5(b,e)). On the other hand, high-magnification HAADF-STEM images show arrays of TDs with an in-plane Burger’s vector, forming low-angle grain boundaries (Fig. 5(c,f)). This observation provides further confirmation that the disorder between grains in the films is indeed dominated by the in-plane twist. Strong strain contrast around these boundaries even in HAADF-STEM images implicates the existence of a high level of microstrain, in agreement with the SAED results from above. Different from the previous reports on BSO thin films^15,22^, Ruddlesden-Popper faults were rarely observed in the films.

**Figure 6:**
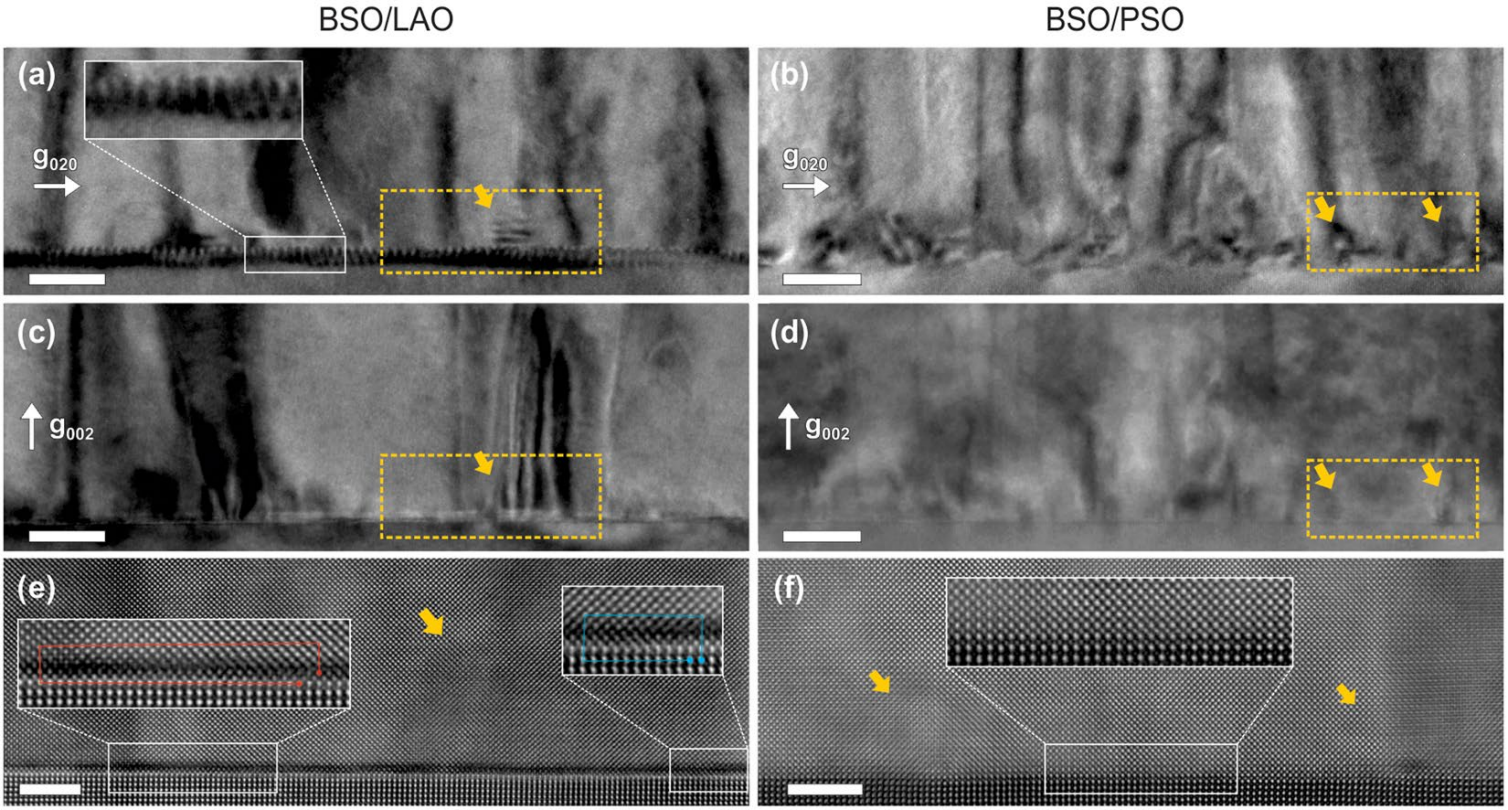
Cross-sectional TBBF-TEM and HAADF-STEM images of the film-substrate interface: (left column) BSO on LAO and (right column) BSO on PSO. (**a–d**) TBBF images with reflection (**a**,**b**) **g** = 020 and (**c**,**d**) **g** = 002. Images were obtained from the same regions. Inset in (**a**) is the magnified area with periodic contrast. (**e** and **f**) High-magnification HAADF-STEM images from regions inside the yellow dotted boxes in (**a**–**d**). Magnified images of the interface are shown as insets. In (**e**), two different types of dislocations are shown. The red Burger’s circuit illustrates the presence of a dislocation with **b** = a_BSO_[001] and two MDs with **b** = a_BSO_[010]. The blue Burger’s circuit illustrates a MD with **b** = a_BSO_[010]. Misoriented islands are marked by the arrows. Scale bars are 20 nm in (**a**–**d**) and 5 nm in (**e**,**f**).

The difference in microstructure between BSO films grown on different substrates can be ascribed to the lattice mismatch between the BSO and the substrate. The lattice mismatch affects the interfacial atomic structure, which subsequently affects the microstructure of the film. To compare the interface structure of these two films, two-beam bright-field (TBBF) images of BSO on LAO and PSO were acquired (see Fig. 6(a–d)). The TBBF images with **g** = 020 reveal clear differences between the two films at the film-substrate interface. While BSO on LAO shows periodic contrast modulation at the interface (as shown in the inset of Fig. 6(a)), BSO on PSO displays no such contrast. This periodic contrast originates from the strain around MDs at the interface^22,26^. The periodicity of the contrast is half of the distance between MDs as the two-beam condition with **g** = 020 generates two contrast flips per MD with **b** = a_BSO_[010]^20,33^. The larger lattice mismatch between BSO and LAO leads to a considerably higher density of MDs at the interface, resulting in the observed contrast. The distance between MDs with **b** = a_BSO_[010] was determined to be 4.7 ± 2.5 nm (11.3 ± 0.6 u.c._BSO_), which is consistent with the prediction of 4.8 nm (12 u.c._BSO_) based on the lattice parameter measured via XRD. TBBF images with **g** = 002 from the same regions were also recorded (Fig. 6(c,d)). As expected, the MDs are not visible in these images, due to the invisibility criterion of **g·b** = 0. The TBDF images with **g** = 020 also reveal the presence of small islands at the interfaces in both films, as marked by the yellow arrows. Even though it is unclear why the islands are formed, they are correlated with the in-plane misorientations of the grains, because they are visible in the TBBF images with **g** = 020 and not with **g** = 002.

Figure 6(e,f) show atomic-resolution HAADF-STEM images acquired from the highlighted regions in Fig. 6(a,b), respectively. The BSO-LAO interface exhibits a distinct dark contrast, which is clearly seen on the BSO side, immediately above the atomically-sharp interface (see Fig. 6(e)). A high density of MDs formed at this interface in both [100] and [010] directions is most likely the origin of this dark contrast. One of these typical MDs, with a Burger’s vector of **b** = a_BSO_[010], is shown using the blue Burger’s circuit in Fig. 6(e). In addition to these typical MDs, dislocations with **b** = a_BSO_[001] were also observed, as exemplified by the red Burger’s circuit, which is drawn over a large area because the dislocation was inclined with respect to the electron beam. Such unusual dislocations have been reported at other perovskite-perovskite interfaces, such as PbTiO_3_–LaAlO_3_(111)^26^ and BiFeO_3_–LaAlO_3_(001)^27^. The dislocations with **b** = a_BSO_[001] are accompanied by inclination of (001) planes, meaning also out-of-plane tilt of the grains in the film. The presence of these dislocations with **b** = a_BSO_[001] thus explains the large out-of-plane tilt observed in BSO on LAO. Quantitatively, one dislocation with **b** = a_BSO_[001] in a 30 nm-wide grain corresponds to an out-of-plane tilt of about 0.8°, which is in decent agreement with the ω X-ray RC result. The interface of BSO on PSO in Fig. 6(f) is MD-free because no MDs could be found in the field-of-view of this area. The measured distance between MDs (with **b** = a_BSO_[010]) was 20.0 ± 6.0 nm (48.5 ± 14.5 u.c._BSO_), which is consistent with the expected distance of 16.6 nm (40 u.c._BSO_) based on lattice parameters measured via XRD. The relatively sharp interface and low dislocation density account for the smaller amount of in-plane twist compared to BSO on LAO, and thus insignificant out-of-plane tilt. Again, this is consistent with the XRD results. One issue that remains to be clarified from the above analysis is why BSO films grown on these two substrates have such similar mobilities in these conditions. Sensitivity of electron scattering to out-of-plane tilt is apparently not particularly high. Instead, we consider that the density of low-angle in-plane grain boundaries is a dominating factor limiting electron mobility, as it is similar in both films.

**Figure 7:**
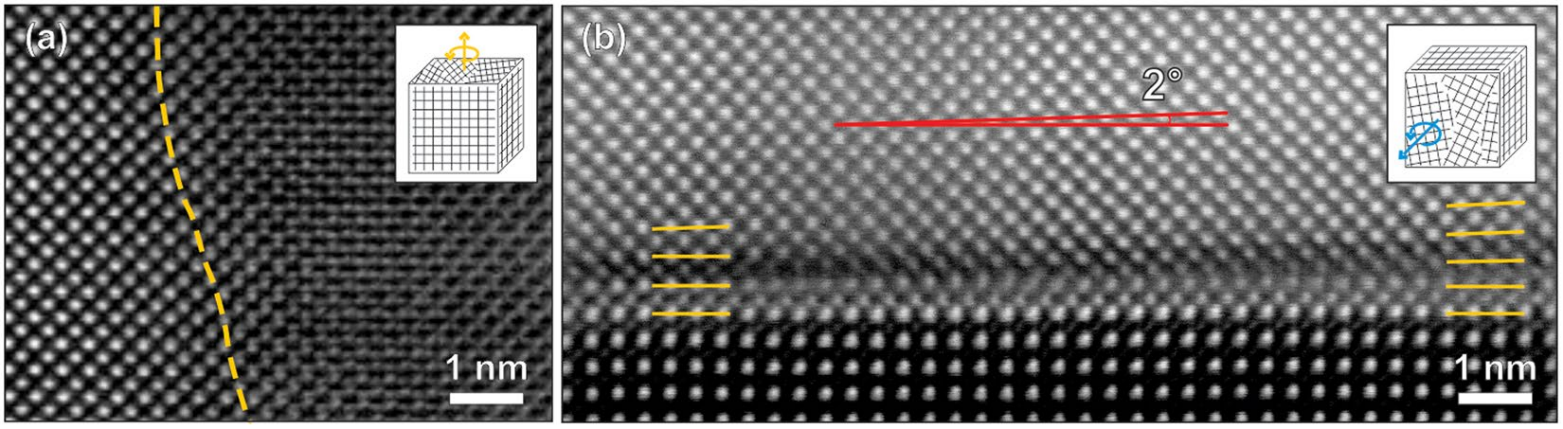
High-resolution ADF-STEM images of BSO on LAO showing (**a**) in-plane twist and (**b**) out-of-plane tilt. A grain boundary is approximately marked with the yellow dashed line in (**a**). In (**b**), (001) planes near the interface are marked with yellow lines, and the out-of-plane tilt angle (about 2 degrees) of BSO (001) planes with respect to LAO (001) planes is shown with the red lines. The insets show a simple model of each form of rotational disorder.

These small in-plane twists and out-of-plane tilts in some cases can be directly visualized by high-resolution ADF-STEM images as shown in Fig. 7. Figure 7(a) shows a boundary of two grains with different in-plane orientations; while the left-hand-side grain shows clear atomic columns, the right-hand-side grain exhibits elongated patterns due to the in-plane twist. An example of the out-of-plane tilt is captured in Fig. 7(b), where approximately 2° inclination of BSO (001) planes with respect to LAO (001) planes can be seen.

In summary, the crystal structure of La-doped BSO films grown on two different substrates, LAO and PSO, were investigated using various TEM techniques and the results, wherever possible, were compared with XRD data. Due to considerably larger lattice mismatch between a BSO film and a LAO substrate (8.7%), the BSO-LAO interface contains a high density (2.2 × 10^6^ cm^−1^) of MDs with **b** = a_BSO_[100] and **b** = a_BSO_[010]. This interface also contains dislocations with **b** = a_BSO_[001], which result in out-of-plane grain tilt in BSO films. The BSO-PSO interface, on the other hand, has a lattice mismatch of only 2.4% between the film and the substrate, and exhibits a lower density (5.0 × 10^5^ cm^−1^) of MDs with **b** = a_BSO_[100] and **b** = a_BSO_[010] only. These differences in interface atomic structures engender different resulting microstructures of the two films, even though both films have a similar columnar structure with grain widths of about 31 nm. Using TBDF imaging, grains with in-plane twist were observed in both films, whereas grains with out-of-plane tilt were observed only in BSO on LAO. From SAED analysis, the in-plane twist was evaluated to be 1.8° ± 0.3° and 1.4° ± 0.2° for BSO on LAO and PSO, respectively. In addition, arrays of TDs forming low-angle grain boundaries between these predominantly in-plane twisted grains were observed from atomic-resolution plan-view ADF-STEM images. Using this analysis as an example, it is shown that TEM-based analysis can provide detailed information about the microstructure including grain sizes and orientations, dislocations types and their density/location, and presence of local strain in such films. When such analysis is combined with complimentary XRD measurements, complete structural characterization of the entire films can be accomplished.
